# Two new species of *Phylloporus* (Fungi, Boletales) from tropical *Quercus* forests in eastern Mexico

**DOI:** 10.3897/mycokeys.51.33529

**Published:** 2019-05-08

**Authors:** Leticia Montoya, Edith Garay-Serrano, Victor M. Bandala

**Affiliations:** 1 Red Biodiversidad y Sistemática, Instituto de Ecología A.C., P.O. Box 63, Xalapa, Veracruz, 91000, México Instituto de Ecología A.C. Xalapa Mexico

**Keywords:** ectomycorrhizal fungi, Neotropical fungi, oak forest

## Abstract

We present a proposal of two new species of *Phylloporus* discovered in tropical oak forests from central Veracruz, Mexico. Both species were distinguished based on macro and micro-morphologic features and supported with a molecular phylogenetic analysis, based on sequences of nuc rDNA ITS, D1, D2 and D3 domains of nuc 28S rDNA (LSU), and transcription elongation factor 1-alpha (tef-1α). In the phylogenetic reconstruction inferred, the new species clustered in two different clades related to species from USA, Costa Rica and Panama. The recollection of fructifications in monodominant stands of either *Quercusoleoides* or *Q.sapotifolia*, allowed recognizing the distribution of one of the *Phylloporus* species under both *Quercus* species, and the other under *Q.oleoides* only. Detailed macro and microscopic descriptions accompanied by illustrations, photos and a taxonomic discussion are provided.

## Introduction

The genus *Phylloporus* is widely distributed worldwide with approximately 100 species occurring among conifers and broad-leaf trees as potential hosts (Neves 2007; Ortiz-Santana et al. 2007; Neves and Halling 2010; Neves et al. 2010, 2012; Zeng et al. 2013; Ye et al. 2014). Recent research on *Phylloporus* systematics revealed that some species placed under this genus in the past are related to other groups. Such is the case of *P.boletinoides*, that was found to be genetically distant, representing an independent genus, described recently as *Phylloporopsis* (Farid et al. 2018). *Erythrophylloporus* Ming Zhang & T.H. Li recently described, is a lamellate lineage in the Boletaceae, having morphological resemblance to *Phylloporus* (Zhang and Li 2018). Additionally, a high species diversity is being detected in the genus; for example in China, Zeng et al. (2013) recognized at least 21 phylogenetic species of *Phylloporus*, 17 of which represented newly discovered taxa. Most *Phylloporus* species have a tropical and subtropical range of occurrence, although some species, e.g. *P.imbricatus* and *P.pachycystidiatus*, are known to occur in alpine ecosystems (Zeng et al. 2013; Ye et al. 2014). In the Neotropics, an important diversity of *Phylloporus* has been documented since the early works by Singer (1973, 1978) and Singer and Gómez (1984), to more recent contributions by Ortiz et al. (2007), Neves and Halling (2010) and Neves et al. (2012). In the Neotropics, *Quercus*, *Pinus*, *Abies*, *Alnus*, *Dicymbe*, and *Neea*, represent some potential ectomycorrhizal hosts of *Phylloporus* spp. mentioned in the literature (Singer 1978, Montoya et al. 1987; Montoya and Bandala 1991, Ortiz-Santana et al. 2007, Neves 2007, Neves and Halling 2010).

In Mexico, *Phylloporus* has been collected mainly in temperate and mesophytic forests. *Phylloporusguzmanii* Montoya & Bandala, and *P.fagicola* Montoya & Bandala were described as new species, the former found in *Pinus* and *Pinus*-*Quercus* forests, while the latter in mesophyll forest under Fagusgrandifoliavar.mexicana (Montoya and Bandala 1991, 2011). Other records in Mexico correspond to *P.bellus* (Massee) Corner, *P.rhodoxanthus* (Schw.) Bres. (inhabiting *Quercus* and mixed *Pinus*-*Quercus*, *Pinus*-*Abies* forests), *P.centroamericanus* Singer & Gómez and *P.foliiporus* (Murr.) Singer (in *Quercus* and mesophyll forests), P.phaeoxanthusvar.simplex Singer & Gómez and *P.leucomycelinus* (Singer) Singer (in *Quercus* forest) (Singer 1957, 1978; Singer and Gómez 1984; Montoya et al. 1987; Montoya and Bandala 1991; García 1999).

Mexico harbors the greatest center of *Quercus* species diversity with about 160–165 species of the 500–600 known worldwide (Valencia 2004, Nixon 2006; Cavender-Bares 2016). Some species of *Quercus* dominate the canopy of lowland tropical forest relicts in the country (Challenger and Soberón 2008). In the state of Veracruz (eastern Mexico) such forest ecosystems currently cover around 905 km^2^, and are listed by CONABIO as priority terrestrial regions considered Pleistocene relicts (Arriaga et al. 2000). Such tropical *Quercus* forests are seriously fragmented but still shelter populations of diverse biological groups, including endemic species of flora and ectomycorrhizal fungi associated with native *Quercus* trees. Many species of this trophic group of fungi in their tropical range are poorly known in Mexico.

As part of a weekly monitoring of macrofungi in two lowland relicts of tropical *Quercus* forests in eastern Mexico, we have detected, among other ectomycorrhizal fungi, the common presence of *Phylloporus* fructifications. After a macro- and micro-morphological study of the collections, that included molecular phylogenetic analyses based on ITS, LSU and tef-1α sequences, we concluded that the specimens represent two new species inhabiting the tropical *Quercus* forests from eastern Mexico.

## Material and methods

### Sampling and morphological study

A weekly monitoring developed during June-October 2016–2017 in two tropical *Quercus* forests from Central Veracruz (eastern Mexico) were the basis of the present study, including some collections made in 2009 and 2012. The two forests are within private properties, one located at Zentla Co. (850 m alt.) and the other one at Alto Lucero Co. (400–500 m alt.); both forests present monodominant stands of *Q.oleoides* Schltdl. & Cham. and *Q.sapotifolia* Liebm. where the *Phylloporus* samples were gathered.

Macromorphological and color studies of specimens were conducted on different growth stages of fresh material. In the description of each species, alphanumeric nomenclature of colors is based on Kornerup and Wanscher (1967) (e.g. 3A4–5) and Munsell color chart (1994) (e.g. 2.5YR 4/4). Basidiomes were dried in a hot air dehydrator (45 °C) for a week. Measurements and colors of micromorphological structures were recorded in 3% KOH and Melzer´s solution. Thirty five basidiospores per collection were measured in lateral view. Basidiospore sizes are accompanied by the symbols: *X*‒, representing the range of *X* (where *X* is the average of basidiospores length and width in each collection) and *Q*‒ refers to the range of *Q* (where *Q* is the average of the ratio of basidiospore length/basidiospore width in each collection). Line drawings were made under a compound microscope (Nikon Eclipse E400) using an attached drawing tube. Line drawings were made under a compound microscope, using an attached drawing tube. Specimen vouchers are kept at XAL herbarium (Thiers B., continuously updated, Index Herbariorum: http://sweetgum.nybg.org/science/ih/).

### DNA extraction, PCR and sequencing

Genomic DNA was extracted from tissue of dried basidiomes according to Montoya et al. (2014). The ITS region of the nuclear ribosomal RNA gene was amplified using the primers ITS1F/ITS4 (White et al. 1990; Gardes and Bruns 1993), the LSU rRNA gene, D1–D3 domains, using primers LR0R/LR21, NL4, LR5 (Hopple y Vilgalys 1999, O´Donnell 1993, Vilgalys and Hester 1990), and the transcription elongation factor 1-alpha (tef-1α) with primers tef1F/tef1R or EF1-2F/EF1-2R (Morehouse et al. 2003, Zeng et al. 2013). PCR conditions for amplification, and procedures for purification of PCR products follow Montoya et al. (2014) and Herrera et al. (2018). Once sequences were assembled and edited, they were deposited at GenBank (http://www.ncbi.nlm.nih.gov) under accession numbers provided in Table 1.

### Phylogenetic analysis

ITS, LSU and tef-1α sequences of *Phylloporus* generated in this study and sequences of closely related species downloaded after a BLAST search from GenBank database (http://www.ncbi.nlm.nih.gov/), were incorporated in independent datasets (one for each molecular marker) in the PhyDE program v.0.9971 (Müller et al. 2010). Each dataset (TreeBASE accession 23913) was independently aligned on the online Mafft service (Katoh et al. 2017) and inconsistencies were adjusted manually. The best evolutionary model for every dataset was calculated with MEGA 6.06 (Tamura et al. 2013). A concatenated dataset with previously aligned sequences of ITS + LSU + tef-1α was integrated. Maximum Likelihood (ML) analysis for every dataset and concatenated multilocus dataset were performed for phylogenetic inference, with 1000 bootstrap replicates in the same program. Phylogenetic analyses were also performed with MrBayes v 3.2.6 (Ronquist et al. 2012) for 1,000,000 of replicates. The phylogenies from ML and BI analyses were displayed using Mega 6.06 and FigTree v1.4.3 (Rambaut 2016) respectively.

## Results

**Figure 1.**
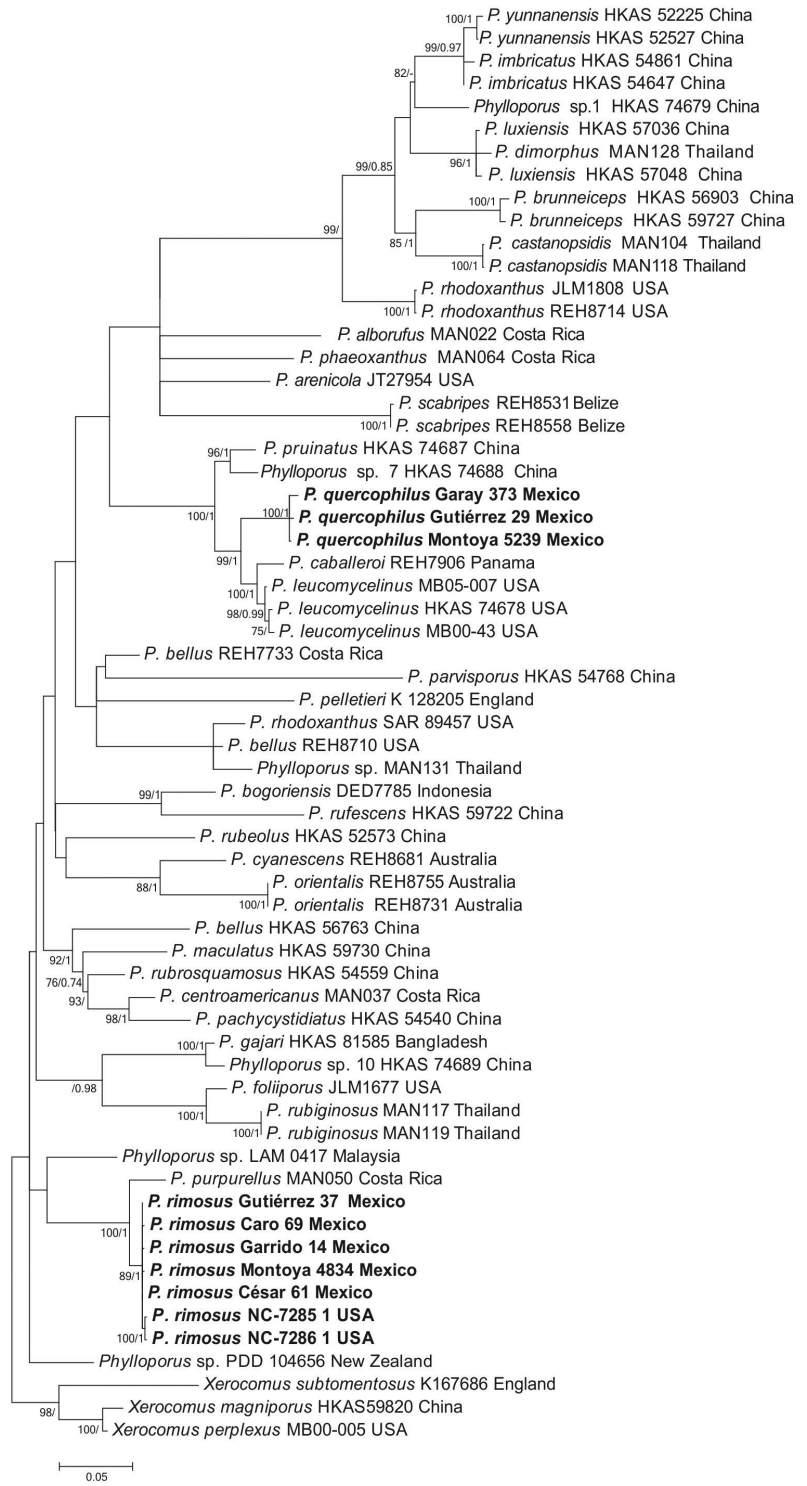
Concatenated three-locus (nrLSU, tef-1α and ITS) phylogenetic analysis by maximum likelihood of *Phylloporus* species. Bootstrap values (BS> 75) / Posterior probabilities (PP > 0.85) are indicated above branches. New species are indicated in bold letters.

Eighteen fresh collections of *Phylloporus* were gathered in the tropical *Quercus* forests studied. Twenty four ITS, LSU and tef-1α sequences (indicated in bold in Table 1) were obtained from eight specimens, and together with 146 sequences of worldwide *Phylloporus* species worldwide were included in the phylogenetic analyses developed (Fig. 1). The best resolution in the phylogenetic analyses was obtained in the combined dataset (nrLSU, tef-1α and ITS). In the individual datasets, both species here described were recognized as independent clades with good BS values. We present here the concatenated three-locus phylogenetic tree (Fig. 1), where the sequences of the Mexican specimens clustered in two strongly supported isolated clades, suggesting that they can be recognized as two different species. Sequences supporting three collections grouped in one clade (BS= 100%, PP= 1.0) sister to sequences of specimens from USA and Panama, identified by Neves et al. (2012) as *P.leucomycelinus* and *P.caballeroi*. Another group of five sequences from Mexican specimens also cluster in a well-supported clade (BS= 89%, PP= 1.0) sister to a sequence identified by those authors as *P.purpurellus* from Costa Rica. Within this latter Mexican clade, sequences recorded as NC 7285-1 and as NC 7286-1, of an unidentified *Phylloporus* species from USA, appear nested in the phylogeny, suggesting that they belong to the same taxon (Fig. 1). Considering the distinctive set of morphological features that the Mexican *Phylloporus* specimens possess (see descriptions below) and with the results of the phylogenetic analysis, we concluded that they represent two new *Phylloporus* species in tropical *Quercus* forests from eastern Mexico and both are proposed here.

**Table 1.** Specimens and sequences considered in this study.

Species	Voucher	Locality	GenBank accession number		
			LSU	ITS	*tef-1*α
* P. alborufus *	MAN022	Costa Rica	JQ003678	JQ003624	–
* P. arenicola *	JT27954	USA	JQ003704	–	–
* P. bellus *	HKAS 56763	China	JQ967196	JQ967239	JQ967153
	REH8710	USA	JQ003686	JQ003618	–
	REH7733	Costa Rica	JQ003661	–	–
* P. bogoriensis *	DED7785	Indonesia	JQ003680	JQ003625	–
* P. brunneiceps *	HKAS 56903	China	JQ967198	JQ967241	JQ967155
	HKAS 59727	China	JQ967201	JQ967244	JQ967158
* P. caballeroi *	REH7906	Panama	JQ003662	JQ003638	–
* P. castanopsidis *	MAN104	Thailand	JQ003689	JQ003642	–
	MAN118	Thailand	JQ003693	JQ003646	–
* P. centroamericanus *	MAN037	Costa Rica	JQ003664	JQ003634	–
* P. cyanescens *	REH8681	Australia	JQ003684	JQ003621	–
* P. dimorphus *	MAN128	Thailand	JQ003697	JQ003648	–
* P. foliiporus *	JLM1677	USA	JQ003687	JQ003641	–
* P. gajari *	HKAS 81585	Bangladesh	KP780423	KP780419	–
* P. imbricatus *	HKAS 54647	China	JQ967202	JQ967245	JQ967159
	HKAS 54861	China	JQ967205	JQ967248	JQ967162
* P. leucomycelinus *	MB05-007	USA	JQ003666	JQ003653	–
	MB00-043	USA	JQ003677	JQ003628	–
	HKAS 74678	USA	JQ967206	JQ967249	JQ967163
* P. luxiensis *	HKAS 57036	China	JQ967207	JQ967250	JQ967164
	HKAS 57048	China	JQ967209	JQ967252	JQ967166
* P. maculatus *	HKAS 59730	China	JQ678698	JQ678696	JQ967194
* P. orientalis *	REH8731	Australia	JQ003700	–	–
	REH8755	Australia	JQ003701	JQ003651	–
* P. pachycystidiatus *	HKAS 54540	China	JQ967211	JQ967254	JQ967168.1
* P. parvisporus *	HKAS 54768	China	JQ967214	JQ967257	JQ967171
* P. pelletieri *	K 128205	England	JQ967215	JQ967258	–
* P. phaeoxanthus *	MAN064	Costa Rica	JQ003670	–	–
* P. purpurellus *	MAN050	Costa Rica	JQ003672	JQ003630	–
*** P. quercophilus ***	Garay 373a	Mexico	MK226557	MK226549	MK314105
	Gutiérrez 29	Mexico	MK226556	MK226548	MK314104
	Montoya 5239	Mexico	MK226558	MK226550	MK314106
* P. rhodoxanthus *	JLM1808	USA	JQ003688	JQ003654	–
	REH8714	USA	JQ003675	JQ003629	–
	SAR 89.457	USA	U11925	–	–
*** P. rimosus ***	Caro 69	Mexico	MK226552	MK226544	
	César 61	Mexico	MK226555	MK226547	
	Garrido14	Mexico	MK226553	MK226545	
	Gutiérrez 37	Mexico	MK226551	MK226543	
	Montoya 4834	Mexico	MK226554	MK226546	
	NC-7285/1	USA	–	AY456356	–
	NC-7286/1	USA	–	AY456355	–
* P. rubeolus *	HKAS 52573	China	JQ967216	JQ967259	JQ967172
* P. rubiginosus *	MAN117	Thailand	JQ003692	JQ003645	–
	MAN119	Thailand	JQ003694	JQ003647	–
* P. rubrosquamosus *	HKAS 54559	China	JQ967219	JQ967262	JQ967175
* P. rufescens *	HKAS 59722	China	JQ967220	JQ967263	JQ967176
* P. scabripes *	REH8531	Belize	JQ003683	JQ003623	–
	REH8558	Belize	–	JQ003622	–
* P. yunnanensis *	HKAS 52225	China	JQ967222	JQ967265	JQ967178
	HKAS 52527	China	JQ967223	JQ967266	JQ967179
*P.* sp. 1	HKAS 74679	China	JQ967228	JQ967271	JQ967184
*P.* sp.10	HKAS 74689	China	JQ967237	JQ967280	JQ967192
* P. pruinatus *	HKAS 74687	China	JQ967235	JQ967278	JQ967190
*P.* sp. 7	HKAS 74688	China	JQ967236	JQ967279	JQ967191
*P.* sp.	LAM 0417	Malaysia	KY091029	–	–
	MAN131	Thailand	JQ003698	JQ003649	–
	PDD 104656	New Zealand	KP191688	–	–
* Xerocomus magniporus *	HKAS 59820	China	JQ678699	JQ678697	JQ967195
* Xerocomus perplexus *	MB00-005	USA	JQ003702	JQ003657	KF030438
* Xerocomus subtomentosus *	K 167686	England	JQ967238	JQ967281	JQ967193

### Description of the new species

#### Phylloporus rimosus

Taxon classificationFungiBoletalesBoletaceae

Bandala, Montoya & Garay sp. nov.

829439

Figs 2a, b , 3 , 4

##### Holotype.

MEXICO. Veracruz: Municipality of Coatepec, Vaquería, gregarious in soil, under *Quercusoleoides* Schltdl. & Cham., 27 June 2012, Montoya 4834 (XAL).

##### Diagnosis.

Recognized by the combination of pileus vinaceous to grayish-vinaceous, surface becoming rimose-areolate with development, the stipe apex with ribbed appearance and scabrous or even with tiny rigid scales and gills staining blue. Its stature (pileus 27–80 mm diam., stipe 27–80 × 7–12 mm), basidiospores and pleurocystidia size and shape, prevents confusion with *P.purpurellus* Singer or with *P.scabripes* B. Ortiz & M.A. Neves.

##### Gene sequences ex-holotype.

MK226546 (ITS), MK226554 (LSU), MK314102 (tef-1α).

##### Etymology.

Referring to the rimose pileus surface.

##### Description.

***Pileus*** 27–80 mm diam, convex to plane-convex, at times faintly depressed at center or even infundibuliform; surface velvety, uniform but frequently rimose-areolate, or fracturing and forming rivulose patches, cracked when seen under lens, vinaceous to grayish-vinaceous (7D4–D5, 7C4; 5YR 3/4, 4/3, 4/4–25Y 6/6), darker in some areas especially towards the margin, or yellowish, reddish-yellow, reddish-brown or even yellowish-beige (10YR 5/4, 6/6) in other parts especially towards the center, some specimens even reddish-vinaceous (7E8–E7) with brownish tinges (7D6–6E8), mature specimens fading to brownish when exposed to the sun; margin slightly incurved, edge entire, at times undulate. ***Lamellae*** subdecurrent to decurrent, 9–15 mm broad, close, bright yellow (3A7, 5A6–A7; 5Y8/8; 4A16), mustard yellow with age (4A6–A7; 4B7–B8), staining blue or greenish-blue when handled, stains becoming reddish or brownish-vinaceous after several minutes, old specimens or specimens long exposed to the sun developing reddish spots at lamellae sides or even dark brownish red or brown at edge; somewhat sinuous when the hymenophore is seen frontally, veined or anastomosed mostly in the area below the pileus and intervenose or even somewhat labyrinthiform, especially when young; lamellullae of different sizes, edge entire. ***Stipe*** 27–80 × 7–12 mm, cylindrical, curved, somewhat sinuous, compact, apex with ribbed appearance by decurrent lines of the lamellae, surface pruinose, scabrous or even with tiny rigid scales, cracked after long exposure to the sun, beige (10YR 6/6–8) or pale yellow (4A/2), or whitish at the bottom of the surface and covered with a reddish or oxide-red (25YR 4/6) pruina, at the middle area reddish-beige (8D16), at times caespitose. ***Basal mycelium*** whitish-cream with some yellow spots or even mustard yellow (5Y8/6). ***Context*** yellow, staining pinkish or pinkish-brown. KOH 3% reddish (10YR 3/6 to 2.5YR 3/4) on pileus, stipe surface and context; NH_4_OH 10% greenish-blue (5Y 2.5/1) on pileus surface, the center of the stain becoming reddish (2.5YR 3/6), brownish at the hymenium, negative in the context and faintly green or negative on stipe surface. ***Odor*** mild to slightly citric. ***Taste*** mild.

***Basidiospores*** (9–) 9.5–14 (–15) × 3.5–5 µm, *X*‒ = 11–12.3 × 4.3–4.6 µm, *Q*‒ = 2.5 2.8 µm, subfusiform, with suprahilar depression, somewhat ventricose, apex attenuated, yellow to amber yellow in KOH, wall slightly thickened (up to 0.5 µm thick). ***Basidia*** 29–50 (–55) × 7–10 (–11) µm, clavate, tetrasporic, rarely trisporic, hyaline, thin walled, unclamped. ***Pleurocystidia*** 42–105 (–120) × 9–27 µm, narrowly to broadly utriform, at times cylindrical or subclavate, rarely sphaeropedunculate (52–58 × 20–23 μm), thin-walled, at times thickened in some areas, some with incrustations, hyaline, abundant, unclamped. ***Cheilocystidia*** (33–) 34–70 (–75) × 8–17 (–19) µm, narrowly utriform, hyaline, thin-walled, at times thickened towards the apex, unclamped. ***Pileipellis*** a trichodermis, with anticlinally oriented hyphae, tightly interwoven, frequently disposed in mounds, hyphae 8–16 µm broad, wall slightly thickened (up to 1 µm), hyaline yellowish-brown; terminal elements 23–64 × 8–14 µm, cylindrical, slightly inflated, other or clavate, pale yellowish-brown. ***Pileus trama*** hyphae 5–16 µm broad, in a lax interwoven arrangement, hyaline, thin walled. ***Hymenophoral trama*** arranged in a more or less regular central strand and somewhat divergent on both sides of the strand, with cylindrical hyphae 7–19 µm broad; some slightly inflated, hyaline, thin-walled, unclamped.

##### Habitat.

In soil, solitary or gregarious, in tropical oak forest, under *Quercusoleoides* and *Q.sapotifolia*.

##### Additional studied material.

MEXICO. Veracruz: Alto Lucero Co., NE Mesa de Venticuatro, 4 Oct 2016, Garrido14; 19 Sep 2017, Gutiérrez 37. Zentla Co. Road Puentecilla-La Piña, 2 July 2009, Ramos 195. Around town of Zentla, 15 June 2016, Montoya 5232a; Montoya 5238; 23 June 2016, Gutiérrez 5, Hervert 84; 30 June 2016, Cesar 61, Hervert 93; 6 July 2016, Caro 69; 30 Aug 2016, Garrido 3; 24 Aug 2017, Garay 368; 7 Sep 2017, César 84 (all at XAL).

**Figure 2.**
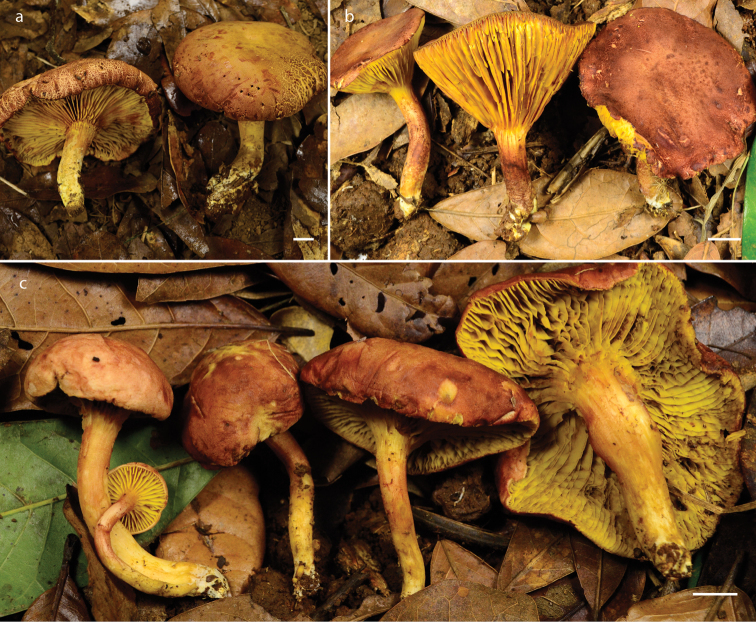
Basidiomes of *Phylloporus* species. **a, b***P.rimosus* (**a** Garrido 3, **b** Montoya 5232a) **c***P.quercophilus* (LM5239 holotype). Scale bars: 10 mm.

**Figure 3.**
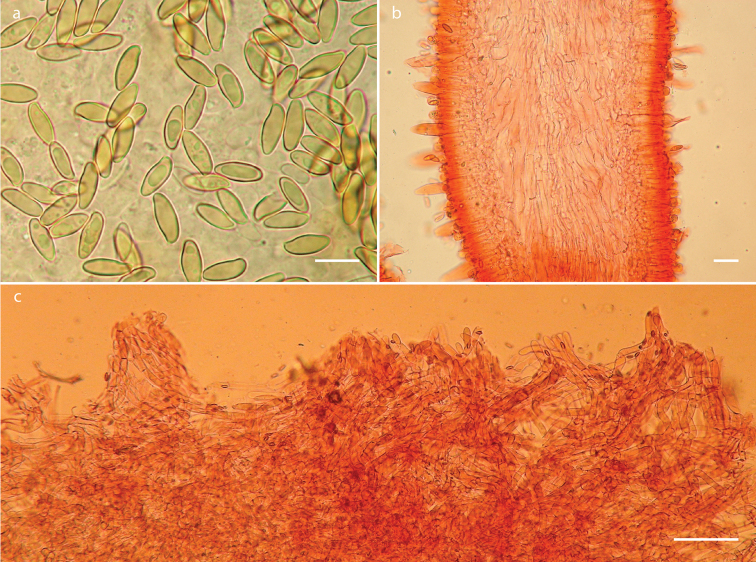
*Phylloporusrimosus* (Montoya 4834, holotype). **a** Basidiospores **b** hymenophoral trama **c** longitudinal section of pileipellis. Scale bars: 10 μm (**a**), 25 μm (**b**), 100 μm (**c**).

**Figure 4.**
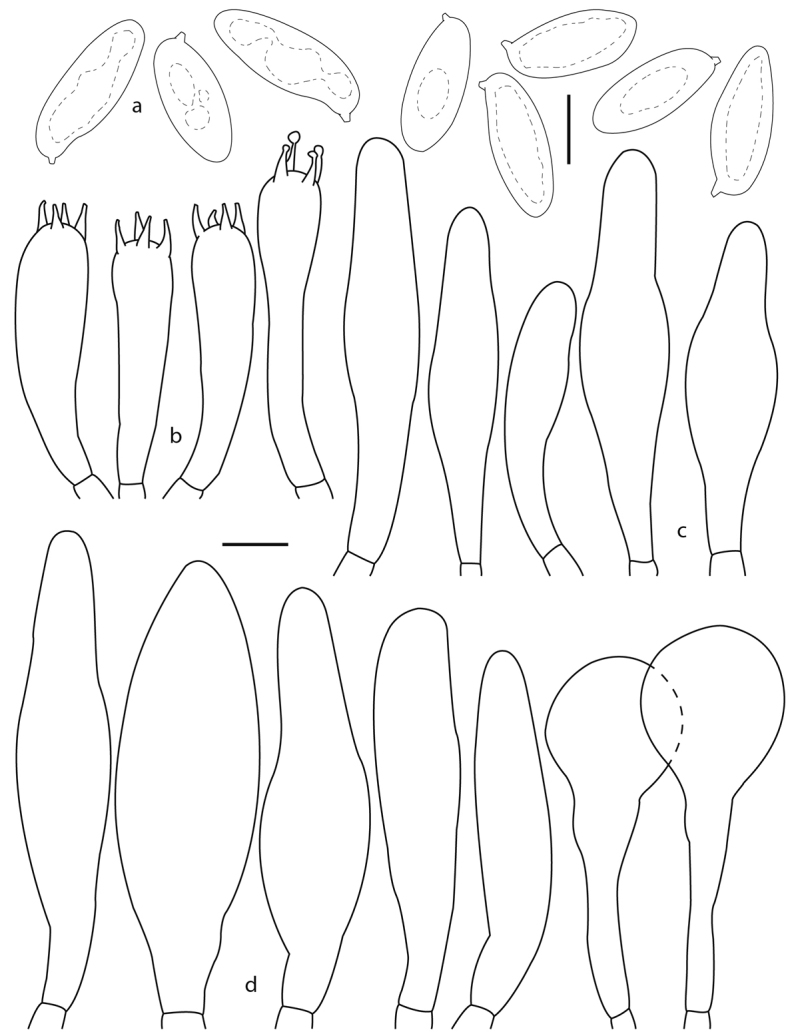
*Phylloporusrimosus* (Montoya 4834, holotype). **a** Basidiospores **b** basidia **c** cheilocystidia **d** pleurocystidia. Scale bars: 5 μm (**a**), 10 μm (**b–d**).

#### Phylloporus quercophilus

Taxon classificationFungiBoletalesBoletaceae

Montoya, Bandala & Garay sp. nov.

829440

Figs 2c , 5 , 6

##### Holotype.

MEXICO. Veracruz: Municipality of Zentla, around town of Zentla, 850 m a.s.l., in soil, in small groups, at tropical oak forest, under *Quercusoleoides* 15 June, 2016, Montoya 5239 (XAL).

##### Diagnosis.

Its reddish pileus tinges together with, context staining reddish, basidiospores 9–13 × 3–4 µm and narrowly utriform or subcylindrical cystidia and its habitat distinguish it from close related species, such as *P.caballeroi* Singer.

##### Gene sequences ex-holotype.

MK226550 (ITS), MK226558 (LSU), MK314106 (tef-1α).

##### Etymology.

In reference to the habitat.

##### Description.

***Pileus*** 15–65 mm diam., hemispheric at first, then becoming convex to plane-convex,; surface velvety, reddish-vinaceous (8D7, 8E7–8), dark reddish-brown (9E6–7), brown (7C5) with pinkish tinges to pinkish-vinaceous (7C6) with paler zones and dark vinaceous tinges (7D6–D7); margin straight to slightly decurved to incurved, undulate. ***Lamellae*** 5–8 mm width, adnate to subdecurrent, close to slightly subdistant, yellow (3A5, 3B7), mustard-yellow (4B7–B8), staining pale brown or blue-greenish when handled, veined or anastomosed mostly below pileus surface and with interparietal veins, margin finely fimbriate, lamellullae of different sizes, with reddish spots. ***Stipe*** 25–55 × 3–13 mm, central, attenuated towards the base, sinuous, compact, reddish-vinaceous (9E7), middle and basal part yellowish to pale brown, bright yellow (3A2, 4A6), with olive to pinkish-vinaceous tinges when young, frequently with a reddish pruina and fine appressed scales over the apex, surface smooth, with peeling fibers especially in mature specimens. ***Basal mycelium*** whitish to yellowish. ***Context*** dirty whitish, staining reddish especially towards the pileus area where it is hygrophanous; stipe at times fistulose but mostly compact, especially at apical area. KOH 3% blackish on pileus, greenish to brown in lamellae, negative in context; NH_4_OH 10% bluish on pileus, or bluish-greenish at the beginning, later blackish in pileus and stipe, dark grayish-blue in context and lamellae. ***Odor*** fruity. ***Taste*** mild.

***Basidiospores*** 9–13 × 3–4 µm, *X*‒ = 10–10.7 × 3.6–3.7 µm, *Q*‒ =2.7–2.9 µm, subcylindrical, with a faint suprahilar depression, attenuated towards apical area and with rounded apex, frontal view subcylindrical, hyaline, with very pale greenish tinges, wall slightly thickened (up to 0.5 µm) 10 to 30% in a field of view dextrinoid. ***Basidia*** 28–42 (–46) × 6–10 µm, clavate, tetrasporic, hyaline, unclamped. ***Pleurocystidia*** 50–102 × 8–16 µm, narrowly utriform, subutriform or irregularly subcylindric, hyaline, pale yellowish, not incrusted, thin walled, at times the wall slightly thickened up to 1 µm, unclamped. ***Cheilocystidia*** 42–90 × 8–14 µm, hyaline, narrowly fusiform to subcylindrical, thin-walled, at times incrusted, unclamped. ***Pileipellis*** a trichodermis composed of more or less erect and tightly interwoven hyphae, at times disposed in mounds, hyphae 7–14 µm broad, thin walled, unclamped; terminal elements 20–48 × 7–14 µm, hyaline, other cells with pale yellow contents, this layer yellowish-brown in KOH at lower magnifications, thin walled, unclamped. ***Pileus trama*** hyphae 6–13 µm broad, in a compact interwoven arrangement, cylindrical to subcylindrical, hyaline, thin walled, at times incrusted in a faintly circumferential striate pattern, unclamped. ***Hymenophoral trama*** divergent; hyphae 6–12 µm broad, thin-walled (< 1 µm thick), at times with resinous like incrustations, some hyphae with a faintly striate appearance, hyaline, unclamped.

##### Habitat.

In soil, in small groups or solitary, in tropical oak forest, under *Quercusoleoides* Schltdl. & Cham.

##### Additional studied material.

MEXICO. Veracruz: Zentla Co., around town of Zentla, 850 m a.s.l., 12 July 2017, Gutiérrez 29; 24 Aug 2017, Garay 366; 7 Sep 2017, Garay 373a (all at XAL).

**Figure 5.**
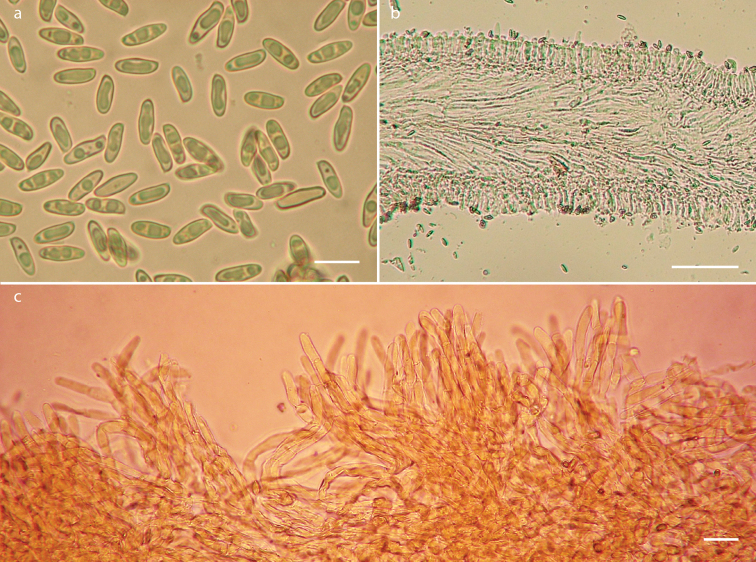
*Phylloporusquercophilus* (Montoya 5239, holotype). **a** Basidiospores **b** hymenophoral trama **c** longitudinal section of pileipellis. Scale bars: 10 μm (**a**), 100 μm (**b**), 25 μm (**c**).

**Figure 6.**
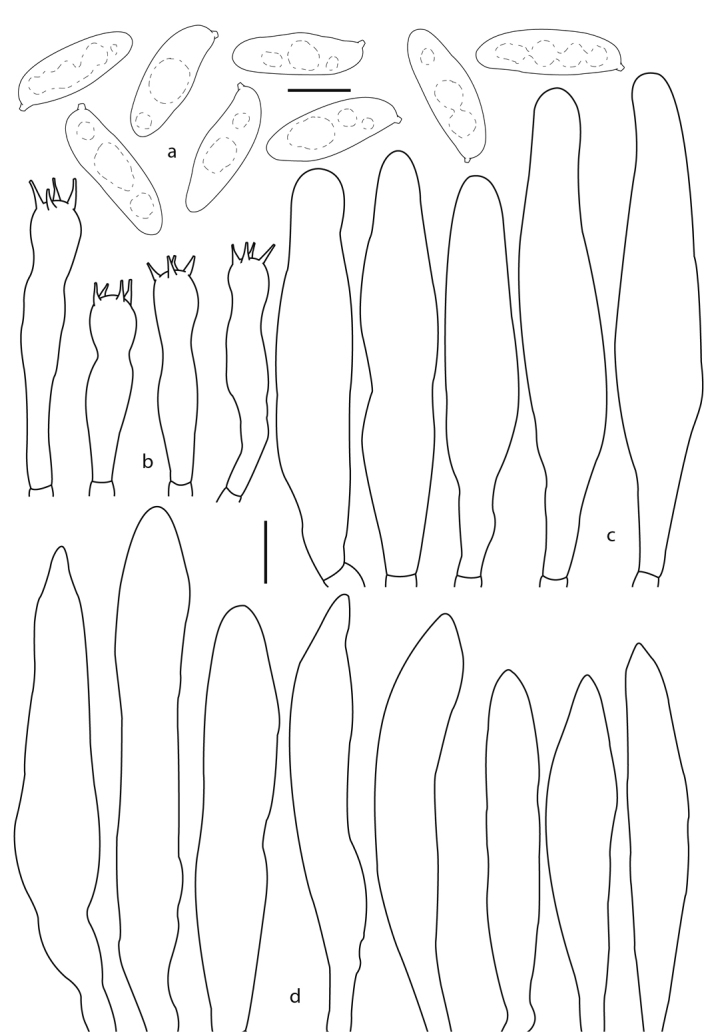
*Phylloporusquercophilus* (Montoya 5239, holotype). **a** Basidiospores **b** basidia **c** pleurocystidia **d** cheilocystidia. Scale bars: 5 μm (**a**), 10 μm (**b–d**).

## Discussion

The multilocus phylogeny inferred demonstrated that *Phylloporusrimosus* and *P.quercophilus* are genetically distant, clustered in separate well-supported clades, and apart from other *Phylloporus* species. Both were found co-habiting in the *Quercus* forests studied. Although they are somewhat similar in their general habit, when comparing the pileus surface, the velvety texture in *P.rimosus* becomes rimose-areolate with development, while in *P.quercophilus* the surface remains uniform. *Phylloporusrimosus* has more robust basidiomes, with a thicker, scabrous and more rigid stipe. The basidiospore sizes, shape and color are different, being larger in *P.rimosus* [(9–) 9.5–14 (–15) × 3.5–5 µm, *X*‒ = 11–12.3 × 4.3–4.6 µm *vs.* 9–12.5 × 3–4 µm, *X*‒ = 10 –10.7 × 3.6–3.7 µm] more ventricose and attenuated towards the apex, and more pigmented, in contrast to *P.quercophilus*. The cystidia appear wider (8–27 µm *vs.* 8–16 µm) and more versiform (including sphaeropedunculate pleurocystidia) in *P.rimosus*. Another difference is that the latter has a hymenophoral trama with the hyphae arranged in a regular central strand and somewhat divergent on both sides, while in *P.quercophilus* that trama is distinctly divergent.

In the phylogenetic analysis (Fig. 1) *P.rimosus* grouped close to a Costa Rican specimen identified as *P.purpurellus* Singer by Neves et al. (2012). According to Singer (1973) the basidiomes of *P.purpurellus* in comparison with the Mexican species, present a tiny habit, with pileus only up to 26 mm diam. and stipe 30 × 4–4.5 mm; shorter basidiospores (7.5–11.3 × 3.3–4 μm) and with cystidia 48–65 × 8.5–12 μm shorter and narrower. In the analysis, the *P.rimosus* clade includes two ITS sequences (NC 7285-1, NC 7286-1) obtained from basidiomes growing in a Loblolly pine (*Pinustaeda*) plantation from North Carolina, USA (after Edwards et al. (2004). Both sequences are inferred to be conspecific with the *P.rimosus* Mexican collections. Loblolly pine is widely distributed in the SE United States (USDA, https://www.fs.fed.us/database/feis/plants/tree/pintae/all.html). Currently, the provenance of our specimens and those of Edwards et al. (2004), reveal that *P.rimosus* displays a range at the eastern portions of both USA and Mexico.

*Phylloporusquercophilus* appeared as a sister species (Fig. 1) to specimens identified by Neves et al. (2012) and Zeng et al. (2013) as *P.caballeroi* Singer and *P.leucomycelinus* Singer. *Phylloporuscaballeroi* described by Singer (1973) from Argentina, differs from *P.quercophilus* by the pileus with olivaceous tinges, lamellae in a closer arrangement, context not staining reddish, and association with *Alnus*. Neves and Halling (2010) offered a broader concept of *P.caballeroi*, and congruent with the original diagnosis, they cite similar basidiospores [4–5 (–6) μm diam. (Q= 2.21)] and ampullaceous cystidia. *Phylloporusleucomycelinus* differs from *P.quercophilus* by the smalller basidiomes (pileus 28–34 mm diam; stipe 27–45 × 3–5 mm), with deep red-brown pileus, lamellae brownish yellow to yellow-brown with olive tinge, and shorter [50–71 × (6–) 11–12] ampullaceous cystidia (Singer 1978).

Considering some morphological and color resemblance, *P.rimosus* and *P.quercophilus* should be compared with *P.scabripes* B. Ortiz and M.A. Neves from Belize, *P.bellus* (Massee) Corner and *P.rufescens* Corner from Singapore (Corner 1970; Singer and Gómez 1984; Ortiz-Santana et al. 2007). However, they are genetically distinct according to the phylogeny inferred here (Fig. 1) [that include sequences produced by Neves et al. (2012) and Zeng et al. (2013)]. *Phylloporusscabripes* is similar to *P.rimosus* because of its distinctly scabrous stipe surface, an unusual feature for a species of *Phylloporus*. The former species differs however, from *P.rimosus* by shorter basidiospores [9.8–12.8 × 3.2–4.8 µm *vs.* (9–) 9.5–14 (–15) × 3.5–5 µm] and shorter and broader pleurocystidia [43.2–80 × 13.6–15.2 µm *vs.* 42–105 (–120) × 9–27 µm)]. The pleurocystidia size also distinguishes *P.scabripes* from *P.quercophilus* (50–102 × 8–16 µm) as also the pileus color of *P.scabripes* [“...pale reddish brown (6D4), paler to tan with age (near 5C4...”] is paler and brownish in range, not as vinaceous, as in both of the Mexican taxa, and the context of *P.scabripes* does not stain reddish. Moreover, *P.scabripes* apparently lacks cheilocystidia.

We concur with Zeng et al. (2013) that the name *P.bellus* has been too widely applied. So we refer here to the original description (Corner 1970) which defines this species with shorter basidiomes than those of *P.rimosus* [stipe (30–40 × 4–10 mm)], with narrower lamellae (4–7 mm width) and shorter basidiospores [8.5–10 × 4.5–5.5 (–6) μm]. The basidiospores of *P.bellus* are even shorter than in *P.quercophilus*. The Asian species *P.bellus* differs also from both Mexican taxa by the context not staining reddish. On the contrary, *P.rufescens* Corner shares with both Mexican taxa the reddening of the context but it finally turns black on exposure (Corner 1970), which does not occur either in *P.rimosus* or in *P.quercophilus*. Other differences among *P.rufescens* and the Mexican species are the shorter size of basidiospores (8–9 × 4–5 μm) and more robust basidiomes (pileus 50–140 mm diam and stipe 25–120 × 6–25 mm).

In Costa Rica, Singer and Gomez (1984) concluded that *Phylloporus* species are present in tropical montane zones forming ectomycorrhiza with *Quercus* spp. and *Alnusjorullensis*. They observed however, that this group of fungi did not occur in lower mountains of the country, and suggested that, it is possibly extremely rare there or perhaps, it is not adapted to *Q.oleoides* or that unknown edaphic or climatic limitations prevent its distribution. Current records of *Phylloporus* in tropical monodominant stands of *Q.oleoides* here described suggest the potential ectomycorrhizal association of *Phylloporus* with this tree species. Additionally, *P.rimosus* represents a first report of *Phylloporus* growing in association with *Q.sapotifolia* trees and even with *Pinustaeda*.

## Supplementary Material

XML Treatment for Phylloporus rimosus

XML Treatment for Phylloporus quercophilus
